# Growth Hormone-Releasing Hormone Receptor Antagonist Modulates Lung Inflammation and Fibrosis due to Bleomycin

**DOI:** 10.1007/s00408-019-00257-w

**Published:** 2019-08-07

**Authors:** Chongxu Zhang, Renzhi Cai, Aaron Lazerson, Gaetan Delcroix, Medhi Wangpaichitr, Mehdi Mirsaeidi, Anthony J. Griswold, Andrew V. Schally, Robert M. Jackson

**Affiliations:** 1 Research Service, Miami VAHS, Miami, FL 33125 USA; 2 Department of Medicine, University of Miami, Miami, FL 33101 USA; 3 Department of Comparative Pathology, University of Miami, Miami, FL 33101 USA; 4 Dr. John T. MacDonald Foundation Department of Human Genetics, University of Miami, Miami, FL 33101 USA; 5 Department of Pathology and Sylvester Cancer Center, University of Miami Miller School of Medicine, Miami, FL 33101 USA; 6 Research Service, Miami VAHS, 1201 NW 16th Street, Miami, FL 33125 USA

**Keywords:** Idiopathic pulmonary fibrosis, Growth hormone-releasing hormone, MIA-602, Bleomycin

## Abstract

**Purpose:**

Growth hormone-releasing hormone (GHRH) is a 44-amino acid peptide that regulates growth hormone (GH) secretion. We hypothesized that a GHRH receptor (GHRH-R) antagonist, MIA-602, would inhibit bleomycin-induced lung inflammation and/or fibrosis in C57Bl/6J mice.

**Methods:**

We tested whether MIA-602 (5 μg or vehicle given subcutaneously [SC] on days 1–21) would decrease lung inflammation (at day 14) and/or fibrosis (at day 28) in mice treated with intraperitoneal (IP) bleomycin (0.8 units on days 1, 3, 7, 10, 14, and 21). Bleomycin resulted in inflammation and fibrosis around airways and vessels evident histologically at days 14 and 28.

**Results:**

Inflammation (histopathologic scores assessed blindly) was visibly less evident in mice treated with MIA-602 for 14 days. After 28 days, lung hydroxyproline (HP) content increased significantly in mice treated with vehicle; in contrast, lung HP did not increase significantly compared to naïve controls in mice treated with GHRH-R antagonist. GHRH-R antagonist increased basal and maximal oxygen consumption of cultured lung fibroblasts. Multiple genes related to chemotaxis, IL-1, chemokines, regulation of inflammation, and extracellular signal–regulated kinases (ERK) were upregulated in lungs of mice treated with bleomycin and MIA-602. MIA-602 also prominently suppressed multiple genes related to the cellular immune response including those for T-cell differentiation, receptor signaling, activation, and cytokine production.

**Conclusions:**

MIA-602 reduced lung inflammation and fibrosis due to bleomycin. Multiple genes related to immune response and T-cell functions were downregulated, supporting the view that MIA-602 can modulate the cellular immune response to bleomycin lung injury.

## Introduction

Idiopathic pulmonary fibrosis (IPF) is the paradigm of fibrosing interstitial lung diseases. It occurs more commonly in aging males, and often has a limited survival time of 3–5 years (median 3.8 years) after diagnosis [1]. Although the disease is of unknown etiology, it is clearly related to specific genetic abnormalities (e.g., *MUC5B*, *SFTPC*, and others) and environmental factors (e.g., dust and smoking) [2]. In response to injury, fibroblasts proliferate and migrate into the lung. They synthesize extracellular matrix, providing a platform for further cellular growth [3]. Myofibroblasts secrete cytokines, such as TGF-ß, with autocrine and paracrine effects that drive fibrosis in the lung [4].

Growth hormone-releasing hormone (GHRH) is secreted primarily from the hypothalamus, but various other tissues can produce it locally [5]. GHRH stimulates the secretion and release of growth hormone (GH) by the pituitary and in turn regulates the secretion of GH and insulin-like growth factor 1 (IGF-1) through the pituitary GH/hepatic IGF-1 axis [6]. We have found expression of pituitary-type GHRH receptor (pGHRH-R) in normal human and IPF lung tissue by western blotting, suggesting that GHRH or GH could participate in lung development, growth, and repair [7].

GHRH belongs to a peptide family that includes glucagon, secretin, vasoactive intestinal peptide (VIP), and pituitary adenylate cyclase-activating peptide (PACAP) [5]. GHRH-R antagonists exert growth-inhibitory effects in cancers in vitro and in vivo [8–10], in addition to having anti-inflammatory and anti-oxidative effects [11].

Human fibroblasts express GHRH receptors, which stimulate proliferation of fibroblasts through GH/IGF-1-mediated signaling. When skin wounds in mice are exposed to GHRH agonist, fibroblasts increase and repair of epithelium is accelerated [12]. GHRH stimulates the expression of α-smooth muscle actin (αSMA), which confers contractile activity in myofibroblasts [13]. In addition to its effects on GH and IGF-1, GHRH-R antagonist MIA-602 inhibits signaling pathways, including PAK1–STAT3/NF-κB in gastric cancer cells, suggesting it could modulate inflammatory and fibrotic processes [14].

We therefore used an established bleomycin model of lung inflammation and fibrosis in C57/Bl6 mice and synthetic GHRH receptor antagonist MIA-602 to test whether inhibition of GHRH receptors would limit inflammation and/or fibrosis.

## Methods

### Materials

The GHRH-R antagonist, MIA-602 (MW 4843), was synthesized (PhAC-Ada, Tyr D-Arg, Asp, Ala, Ile, 5FPhe, Thr, Ala, Har, Tyr(Me), His, Orn, Val, Leu, Abu, Gin, Leu, Ser, Ala, His, Orn, Leu, Leu, Gin, Asp, Ile, Nle, D-Arg, Har NH_2_) at the Miami VAHS by solid phase methods and purified by HPLC.

### Experimental Animals

We used 8-week-old C57Bl/6J male mice (The Jackson Laboratory, Bar Harbor, ME) in these experiments. Mice weighed about 26 g at the start. This protocol was approved by the Miami VAHS Animal Studies Subcommittee (IACUC) and it conformed to PHS Policy. Mice were randomly allocated to the experimental groups and housed in identical filter top cages in a ventilated rack. Mice were exposed to a 12-h light/dark cycle and had free access to standard laboratory chow and water.

### Bleomycin and GHRH-R Antagonist Treatment

All mice were treated with 0.8 units of bleomycin (Bleomycin for Injection USP, Hospira, Lake Forest, IL) intraperitoneally (IP) on days 1, 3, 7, 10, 14, and 21 [15]. Mice (randomly assigned) were simultaneously treated subcutaneously (sc) with MIA-602 (5 μg/day) or its vehicle (100 μL/day) [14]. MIA-602 was dissolved in DMSO (ACS grade; Sigma-Aldrich) and diluted 1:500 in normal saline for daily sc injection on days 0–21.

### Tissue Preparations

Mice were killed by CO_2_ inhalation before any treatment, or on days 14 or 28 for micro-CT scans of lungs and harvesting of lung tissue [16]. The right mainstem bronchus was ligated and the right lung removed and frozen at – 80 °C for hydroxyproline (HP) assays. The left lung was filled with 10% buffered formalin at ~ 25 cm H_2_O pressure, the bronchus ligated, and the lung fixed in formalin. Fixed lungs were embedded in wax and 5 μm sections stained with hematoxylin and eosin (H&E) or Masson’s trichrome stain.

### Lung Histopathology

Fibrosis was quantified in trichrome-stained sections using a modification of the Ashcroft score that describes grades of fibrosis [17]. Ten randomly chosen fields on each slide were scored as described and averaged. Inflammation in lung tissue was quantified in H&E-stained sections.

### Hydroxyproline Assay

Right lungs were weighed and homogenized in 10 volumes of distilled water. Homogenates were hydrolyzed in 12 M HCl at 120 °C for 3 h as previously described [18]. Hydroxyproline was measured colorimetrically (560 nm) after hydrolysis using an assay kit (MAK008, Sigma-Aldrich, St. Louis, MO).

### Micro-CT

Mice in each group were assessed by micro-CT scans (Bruker SkyScan 1176 Low-Dose Micro-CT, Knotich, Belgium) of the lungs after CO_2_ inhalation. A tracheostomy tube was inserted and lungs inflated with air. Scans were examined qualitatively to confirm development of infiltrates and reticular densities.

### Lung Fibroblasts

Newborn mouse lung fibroblasts (Mlg 2908) were obtained from the American Type Culture Collection, Manassas, VA and cultured in Eagle’s minimal essential medium. Cells were grown in 6-well plates before incubation with 1 or 5 µM MIA-602 or vehicle, RNA isolation, and oxygen consumption measurements.

### Annexin V-Propidium Iodide Assay

Apoptosis and necrosis were assessed with annexin V/propidium iodide staining (Annexin V: FITC Assay Kit, Bio Rad, Hercules, CA 94547) of lung fibroblasts incubated in 0, 1, or 5 µM MIA-602 for 24 h [19]. Culture medium and trypsinized cells were collected and centrifuged at 400×*g* for 5 min. The pellet was resuspended in 100 µL annexin V/propidium iodide. The suspension was incubated at 37 °C for 20 min, then washed with PBS, and resuspended in 500 µL PBS and fluorescence quantified by Beckman Coulter Life Sciences CytoFLEX benchtop flow cytometer (Beckman Coulter, Inc., Brea, CA).

### RNA Isolation

RNA was extracted from fixed lung tissue in paraffin blocks using a Quick-RNA™ FFPE Kit (R1008, Zymo Research, Irvine, CA) following the manufacturer’s protocol [20]. Samples were deparaffinized, digested with proteinase K, and decrosslinked at 65 °C for 15 min. RNA lysis buffer was added and mixed with ethanol. The mixtures were transferred to spin columns to isolate total RNA.

RNA was extracted from cultured fibroblasts using a Direct-zol RNA MicroPrep kit (R2060; Zymo Research, Irvine, CA). Cells were washed with PBS and lysed in TRI reagent™, then purified using Direct-zol RNA columns. DNase I treatment was done in columns and RNA eluted in DNase/Rnase-free water.

### Cellular Respiration

We measured effects of MIA-602 on mouse lung fibroblast oxygen consumption using the Agilent Seahorse XF Cell Mito Stress Test (Agilent Technologies, Santa Clara, CA) [21]. Fibroblasts were incubated with vehicle, 1 or 5 µM MIA-602 for 24 h before measurement of oxygen consumption. One day before assay 80,000 fibroblasts were seeded into Seahorse 24-well plates (*n* = 6 wells per condition). Basal respiration was established and oligomycin, FCCP, and rotenone plus antimycin A were added sequentially to measure ATP production, uncoupled respiration, and non-mitochondrial oxygen consumption [22].

### RNA-seq and Pathway Analyses

At least 10 ng of total RNA was used as input for the KAPA RNA HyperPrep Kit with RiboErase (HMR) to create ribosomal RNA-depleted sequencing libraries, including sample indexing, to allow for multiplexing. Cluster generation and sequencing was done on the Illumina cBOT and HiSeq 3000 using reagents provided by Illumina, finally generating > 32 million single-end 100 base reads per sample.

We created de-multiplexed FASTQ files with Illumina supplied scripts in BCL2FASTQ software (v2.17). Illumina adapters were trimmed using the Trim Galore! package and aligned to the mouse reference genome (mm10) with STAR aligner (v2.5.0a) with default alignment parameters [23].

Gene counts were normalized using trimmed mean of M-values (TMM) method. Differential expression between groups was calculated with the exact test implemented in edgeR [24].

Pathway enrichment analyses were completed using Enrichr online and DAVID bioinformatics resource [25, 26].

### Data Analysis

Data are reported as arithmetic means ± SEM or SD as indicated. Medians and 25–75th percentiles were used to describe the distribution of histopathological scores. ANOVA followed by Dunnett’s test or the Bonferroni correction was used for multiple comparisons with a control or among groups [27]. We planned at least six mice available at each time point in each group, so that we would have sufficient power to detect a 20% change in lung hydroxyproline given an assumed coefficient of variance of 0.2. *p* ≤ 0.05 was considered significant.

## Results

### Micro-CT Scans

Mice treated with vehicle developed patchy infiltrative densities that persisted to day 28. Mice that received bleomycin plus MIA-602, the GHRH-R antagonist, appeared to have less prominent infiltrative densities in their lungs.

### Lung Hydroxyproline Contents

**Fig. 1:**
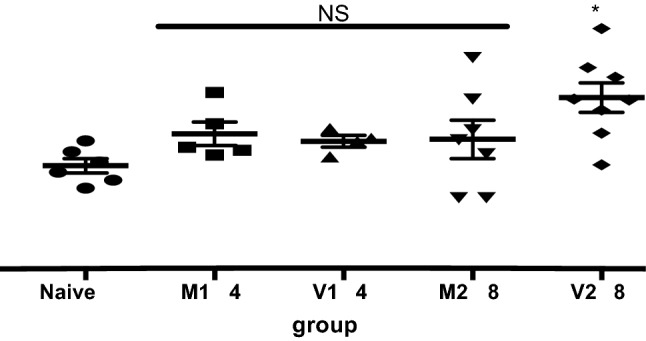
Lung hydroxyproline content. Evaluation of lung hydroxyproline (HP) content to estimate changes in collagen due to bleomycin and the effect of MIA-602. Data shown are mean hydroxyproline contents of right lungs ± SEM at 14- and 28-day time points. Normal C57Bl/6J mice (*n* = 6) had about 20 µg HP in the right lung. HP content increased significantly (*p = 0.0060 compared to Naive) after 28 days in mice treated with bleomycin and vehicle. No significant increase in HP content occurred in lungs of mice treated with bleomycin and the GHRH-R antagonist MIA-602. M14, MIA-602 group at 14 days (*n* = 5); V14, vehicle group at 14 days (*n* = 4); M28, MIA-602 group at 28 days (*n* = 7); V28, vehicle group at 28 days (*n* = 8)

Lung HP content did not increase significantly after 14 days of intermittent treatment with bleomycin in mice receiving MIA-602 or vehicle. However, after 28 days, lung HP content increased significantly in bleomycin-treated mice that received vehicle, but not in bleomycin-treated mice that received MIA-602 on days 1–21. The data are summarized in Fig. 1.

### Lung Histopathology

**Fig. 2:**
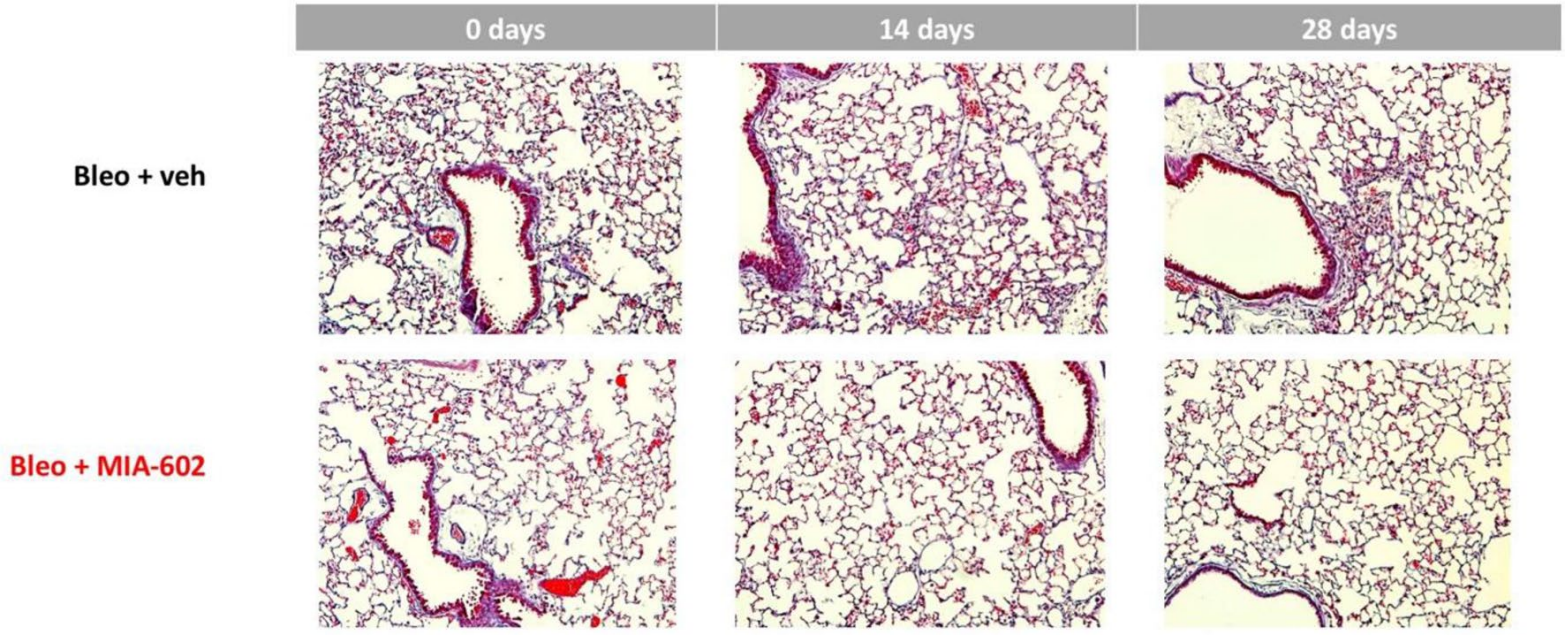
Lung histopathology. Mouse lungs were inflated with buffered formalin to 25 cm H_2_O pressure and fixed. 5 µm sections were stained with Masson’s trichrome stain and assessed semi-quantitatively for inflammation and fibrosis as described in the text. 14 days after bleomycin was started, cellular inflammation and early fibrosis was detected in lungs of mice treated with bleomycin and vehicle. Less inflammation appeared to be present in lungs of mice that received bleomycin and MIA-602 (middle panels). 28 days after bleomycin was started, increased fibrosis was evident in lungs of mice treated with bleomycin and vehicle. Less fibrosis appeared to be present in lungs of mice that received bleomycin and MIA-602, the growth hormone receptor antagonist

After 14 days of intermittent bleomycin, inflammation and patchy mild fibrosis were qualitatively evident in mouse lungs. More extensive fibrosis and fewer inflammatory cells were evident after 28 days. Both inflammatory changes and fibrosis appeared decreased in mice that received MIA-602 in addition to bleomycin. Representative examples are shown in Fig. 2.

MIA-602 appeared to reduce inflammation after 14 days in lungs of bleomycin-treated mice (0.9 [median]; 0.6–1.2 [25–75th percentile]; *n* = 4), compared to mice receiving vehicle (1.4; 0.9–1.8; *n* = 4). MIA-602 appeared to reduce fibrosis after 28 days in lungs of bleomycin-treated mice that received MIA-602 (2.0; 1.4–2.6; *n* = 5), but not in mice treated with vehicle (2.2; 2.1–2.9; *n* = 8).

### Lung Fibroblast Response to MIA-602

Lung fibroblasts were exposed to MIA-602 or vehicle in vitro for 24 h before annexin V/PI assay. MIA-602 caused predominantly cytolytic cell death (1 µM, 9.3 ± 1.1%; 5 µM, 34.9 ± 2.5%; *p* = 0.0002) rather than apoptosis.

### Cellular Respiration

**Fig. 3:**
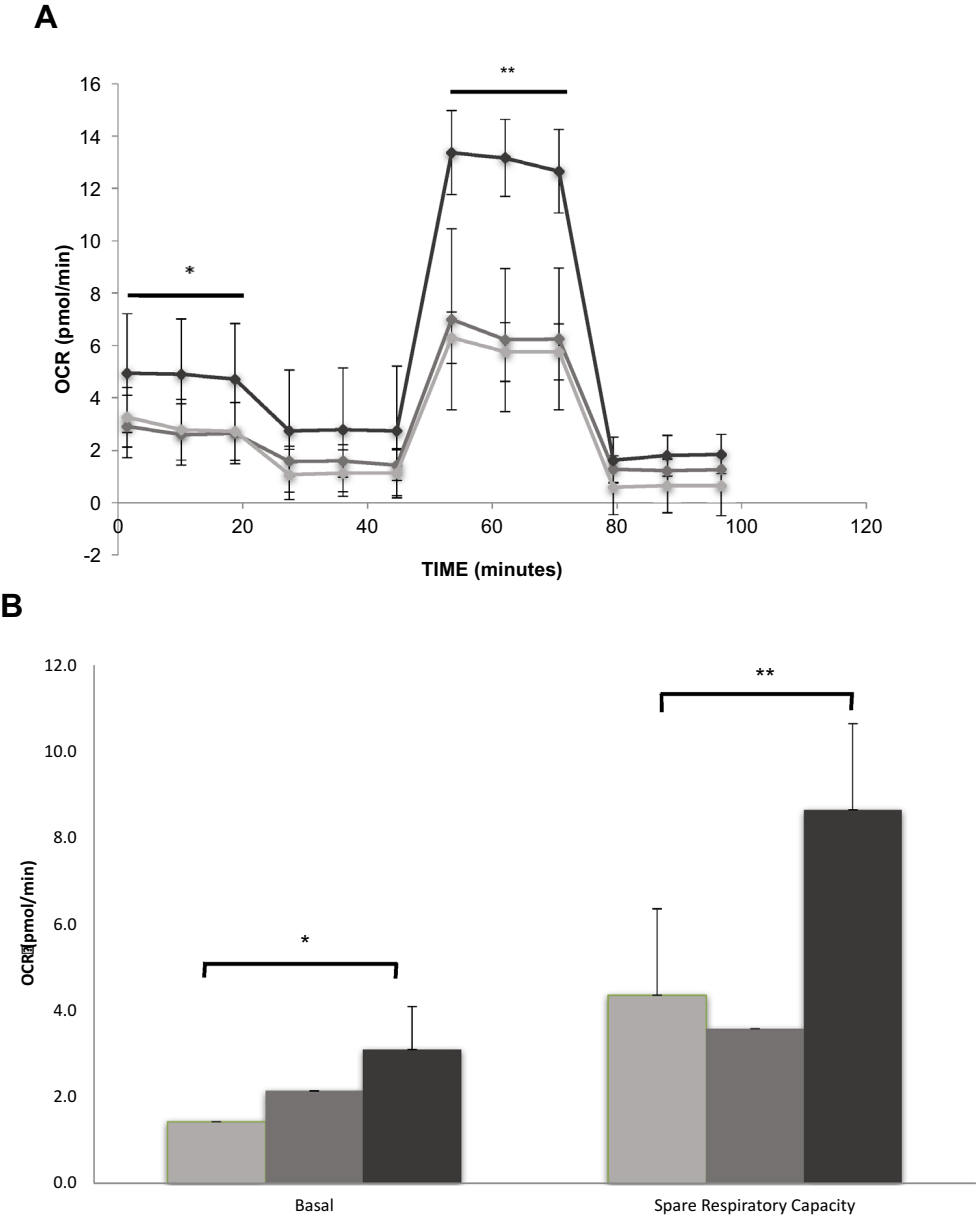
GHRH-R antagonist increases lung fibroblast basal and maximal oxygen consumption. Panel A shows a representative mitochondrial stress assay of mouse lung fibroblasts (data shown are means ± SD of 6 wells in each condition) exposed to vehicle (light gray), 1 µM (medium gray) or 5 µM (black) MIA-602 for 24 h before measurements of oxygen consumption with oligomycin, FCCP, antimycin A, and rotenone. 5 µM MIA-602 increased basal oxygen consumption (*p = 0.0403 compared to vehicle) and maximal uncoupled respiration (**p < 0.0001) of normal mouse lung fibroblasts. Panel B shows that 5 μM MIA-602 increased both basal respiration (*p = 0.0125) and spare respiratory capacity (**p < 0.0001)

In vitro, MIA-602 at 5 µM concentration in complete medium increased fibroblast basal respiration and maximal respiration (after FCCP), compared to vehicle. Both 1 and 5 µM MIA-602 also appeared to increase non-mitochondrial respiration (after antimycin A and rotenone). These data are summarized in Fig. 3.

### RNA-seq Gene Expression

We explored the effects of MIA-602 or vehicle on gene expression in lungs from mice treated in vivo with bleomycin for 28 days (data not shown). Several physiologically relevant genes were expressed differently after treatment with bleomycin (absolute fold change > 1.5 and FDR < 0.01). Specifically, after 28 days during which bleomycin was administered, genes related to the extracellular matrix, Wnt regulation and signaling, and the extracellular region were upregulated, consistent with known effects of bleomycin. We found several relevant genes to be downregulated by bleomycin, including those related to lung morphogenesis and development, extracellular matrix organization, and alveolar septal development.

**Fig. 4:**
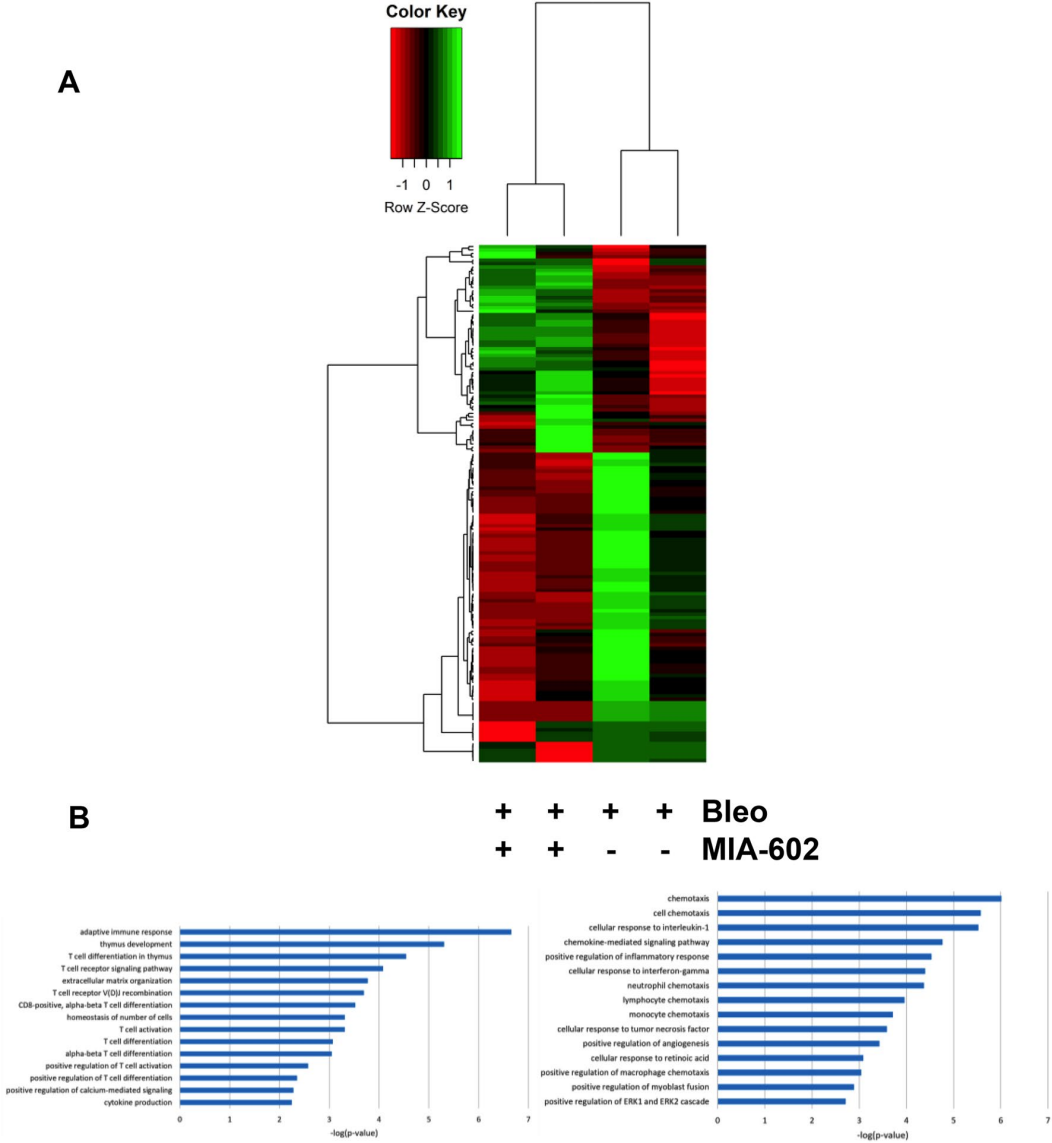
Transcriptomic analysis of mouse lung tissue RNA after treatment of mice in vivo with bleomycin and MIA-602 or vehicle. Panel **a** Heat map analysis showing differential gene expression in lungs from mice treated with bleomycin for 28 days that also received MIA-602 (+) or vehicle (−) for the first 21 days. The left two columns show gene expression in bleomycin- and MIA-602-treated mice, whereas the right two columns show gene expression in bleomycin- and vehicle-treated lungs. Panel **b** Pathway analysis showing differentially expressed genes (downregulated, left panel; upregulated, right panel) in lungs from bleomycin-treated mice also treated with MIA-602 compared to those also treated with vehicle

Transcriptome profiles then showed numerous genes expressed differently after treatment with MIA-602. Inversely modulated genes were highly enriched in pathways related to the adaptive immune response, T-cell differentiation, T-cell signaling, extracellular matrix organization, T-cell activation and differentiation, and cytokine production, all consistent with the putative anti-inflammatory and anti-fibrotic effects of MIA-602. The ten most differentially expressed genes detected in lung tissue treated with MIA-602 compared to vehicle treatment are shown in Table 1. Those genes differentially expressed in fibrotic lungs from bleomycin-exposed mice treated with MIA-602 compared to vehicle are displayed as a heat map in Fig. 4.

**Table 1:** Clusters of the differentially expressed genes and their ontology between bleomycin-induced pulmonary fibrosis treated vs. untreated with MIA-602

Name	Gene list	*p* value	Adjusted *p* value
T-cell differentiation (GO:0030217)	CD4, CD8A, RAG2, RAG1	0.000003374	0.0007392
T-cell activation (GO:0042110)	CD4, CD8A, CD3G, CD3E, LAT	0.000003777	0.0007392
T-cell receptor signaling pathway (GO:0050852)	PSMB11, CD4, THEMIS, CD3G, CD3E, LAT	0.000004630	0.0007392
Antigen receptor-mediated signaling pathway (GO:0050851)	PSMB11, CD4, THEMIS, CD3E, LAT	0.00006007	0.007193
V(D) J recombination (GO:0033151)	RAG2, RAG1	0.0001910	0.01829
Enzyme-linked receptor protein signaling pathway (GO:0007167)	CD4, CD8A, NPPA, CD3A	0.0002969	0.02370
T-cell differentiation in thymus (GO:0033077)	RAG2, RAG1	0.0004471	0.02677
Regulation of leukocyte cell–cell adhesion (GO:1903037)	CD4, LAT	0.0004471	0.02677
Regulation of lymphocyte activation (GO:0051249)	CD4. LAT	0.0006144	0.03270
Lymphocyte differentiation (GO:0030098)	CD4, RAG2, RAG1	0.0009974	0.04777

Similarly, we directly tested the in vitro effects of 1 and 5 µM MIA-602 on normal mouse lung fibroblasts (not exposed to bleomycin) and found significant downregulation of genes involved in collagen fibril organization, cell–matrix adhesion, and elastic fiber assembly, all consistent with the demonstrated anti-fibrotic effects of MIA-602. Upregulated fibroblast genes included those related to protein kinase activity, the JAK-STAT cascade, cell cycle, and DNA replication including histones.

## Discussion

Pathophysiological GH secretion and IGF-1 activation have growth-promoting effects in the lung resulting in increased alveolar size [28]. IGF-1 itself increases α-smooth muscle actin in lung fibroblasts and promotes a myofibroblast phenotype. The pituitary-type GHRH receptor is present in both normal and IPF lung tissue [7], suggesting that local secretion of GH may occur physiologically and have direct effects on lung tissue.

MIA-602 partially inhibits both lung inflammation and fibrosis, assessed histopathologically and biochemically, after intraperitoneal bleomycin. RNA-seq data, importantly, show suppression of the adaptive immune response, T-cell differentiation and activation, and cytokine production by MIA-602 in bleomycin-treated mouse lungs. The effects of the GHRH-R antagonist we observe have implications for fibrosing lung diseases in humans, and they could, importantly, reveal novel pathways amenable to clinical drug development [29].

We initially confirmed development of lung infiltrates due to bleomycin with micro-CT scans, inflammation, and fibrosis with histopathological examination, and increased collagen with biochemical assays. Similar models have been used successfully in the development of anti-fibrotic drugs, including pirfenidone and nintedanib.

Senescent fibroblasts that display respiratory abnormalities, indicating mitochondrial damage, express both STAT3 and p21 as markers of the senescent phenotype [30]. MIA-602 downregulates p21-activated kinase and STAT3 and NFκB in gastric cancer cells [14]. GHRH antagonists like MIA-602 could modulate the senescent phenotype leading to fibrosis, and conceivably be one of the mechanisms that lessens the fibrotic response in this model.

In vitro, MIA-602 at micromolar concentrations increased basal and maximal mitochondrial respiration, and it resulted in marked cytolytic death of mouse lung fibroblasts. Mitochondrial dysfunction and loss of apoptotic potential occur in fibroblasts from IPF lungs, and enhancement of mitochondrial function itself by MIA-602 at lower concentrations in vivo might modulate fibrosis by maintaining the capacity for mitophagy and apoptosis [31].

GHRH-R antagonists decrease lipid peroxidation, protein carbonyls, and nitrotyrosine in prostate cancer cells [32], indicating antioxidant effects that would augment their other anti-inflammatory effects [33]. Both GH and IGF-1 stimulate neutrophil superoxide (O_2_^−^) production [9]. Since GHRH-R antagonists inhibit GH secretion and IGF-1 activation [34], it could be predicted that they would inhibit both O_2_^−^ and hydrogen peroxide (H_2_O_2_) release during the inflammatory phase of injury [35]. Cellular levels of oxidant stress are decreased by MIA-602, and its antioxidant effect would limit redox signaling in response to receptor ligation [11].

MIA-602 disrupts the PI3K/AKT pathway in several experimental systems. PI3K/AKT signaling is involved in the pathogenesis of bleomycin-induced fibrosis [36], and suppression of the PI3K/AKT pathway by GHRH antagonist could also lessen lung fibrosis. GHRH clearly appears involved in the lung’s response to treatment with bleomycin and subsequent healing.

Genes related to the extracellular matrix and the Wnt pathway were over expressed in fibrotic lungs from mice treated with bleomycin, compared to gene expression in naïve controls [37]. In contrast, genes related to epithelial tube branching, lung morphogenesis, and lung development were under expressed after bleomycin and development of fibrosis. Genes related the immune response, cellular adhesion, and remodeling, and T-cell signaling were also found to be upregulated in lungs from rats treated with TGF-ß [38].

T cells play a central role in the lungs of patients with pulmonary fibrosis [39]. T-cell receptors and costimulatory molecules are required for activation of T cells and in development of inflammation-driven lung fibrosis [40]. We found that downregulation of T-cell receptor complex genes (CD3E, CD3G, CD4, and CD8A) had the highest associations in pathway analyses. MIA-602 may thus play an important role in lung tissue by modification of T-cell signaling and potentially reducing inflammation and fibrosis.

These data show that GHRH-R is present in human lungs; and, in a relevant in vivo model, lung fibrosis is modulated by its inhibition. Functional findings implicate GHRH and GH in fibrosing lung disease, and they are consistent with demonstrated effects of the GHRH-R antagonist on mitochondrial respiration and fibroblast cytotoxicity. MIA-602 inhibits intracellular signaling pathways, including p21-activated kinase/STAT3/NFκB and PI3K/AKT, in addition to having intrinsic antioxidant activity. Further, it could support mitochondrial function and maintain autophagy, minimizing fibrosis. These findings suggest the merit of further investigations of GHRH-R antagonists like MIA-602 regarding their mechanisms of action and applications in management of pulmonary fibrosis.
